# Post-transplant Lymphoproliferative Disorder (PTLD) in the US Population: Demographics, Treatment Characteristics, and Survival Analysis

**DOI:** 10.7759/cureus.39777

**Published:** 2023-05-31

**Authors:** Asad Ullah, Kue T Lee, Kali Malham, Abdul Qahar Khan Yasinzai, Imran Khan, Bina Asif, Abdul Waheed, Saleh Heneidi, Nabin R Karki, Feroze Sidhwa

**Affiliations:** 1 Pathology, Vanderbilt University Medical Center, Augusta, USA; 2 ENT, Medical College of Georgia, Augusta, USA; 3 Surgery, Medical College of Georgia, Augusta, USA; 4 Surgery, Bolan Medical College, Quetta, PAK; 5 Medicine, Bannu Medical College, Bannu, PAK; 6 Surgery, San Joaquin General Hospital, French Camp, USA; 7 Pathology, Cedars-Sinai Medical Center, Los Angeles, USA; 8 Oncology, Mitchell Cancer Institute, University of South Alabama, Mobile, USA; 9 General Surgery/Trauma and Critical Care, San Joaquin General Hospital, French Camp, USA

**Keywords:** outcomes, prognosis, seer database, immunosuppression, post-transplant lymphoproliferative disorder

## Abstract

Background: Post-transplant lymphoproliferative disorder (PTLD) is a lymphoplasmacytic proliferative disorder in the setting of hematopoietic stem cells and solid organ transplants. PTLD is divided into nondestructive, polymorphic, monomorphic, and classical Hodgkin lymphoma subtypes. Most cases of PTLDs are Epstein-Barr virus (EBV) related (two third of the cases), and most are of B cell (80-85%) origin. The polymorphic PTLD subtype can be locally destructive and show malignant features. Treatment for PTLD includes a reduction in immunosuppression, surgery, cytotoxic chemotherapy and/or immunotherapy, anti-viral agents, and/or radiation. The aim of this study was to examine the demographic factors and treatment modalities that influence survival in patients with polymorphic PTLD.

Methods: About 332 cases of polymorphic PTLD were identified from 2000 to 2018 using the Surveillance, Epidemiology, and End Results (SEER) database.

Results: The median age of the patients was found to be 44 years. The most common age groups were between the ages of 1-19 years (n=100. 30.1%) and 60-69 years (n=70. 21.1%). The majority of cases in this cohort underwent systemic (cytotoxic chemo and/or immuno) therapy only (n=137, 41.3%), while 129 (38.9%) cases did not undergo any treatment. The overall five-year observed survival was 54.6% (95% confidence interval (CI), 51.1 - 58.1). One-year and five-year survival with systemic therapy was 63.8% (95% CI, 59.6 - 68.0) and 52.5% (95% CI, 47.7 - 57.3), respectively. The one-year and five-year survival with surgery was 87.3% (95% CI, 81.2-93.4) and 60.8% (95% CI., 42.2 - 79.4), respectively. The one-year and five-year without therapy were 67.6% (95% CI, 63.2-72.0) and 49.6% (95% CI, 43.5-55.7), respectively. Univariate analysis revealed that surgery alone (hazard ratio (HR) 0.386 (0.170-0.879), p = 0.023) was a positive predictor of survival. Race and sex were not predictors of survival, although age >55 years was a negative predictor for survival (HR 1.128 (1.139-1.346), p <0.001).

Conclusion: Polymorphic PTLD is a destructive complication of organ transplantation that is usually associated with EBV positivity. We found that it most often presents in the pediatric age group, and its occurrence in those older than 55 years was associated with a worse prognosis. Treatment with surgery alone is associated with improved outcomes and should be considered in addition to a reduction in immunosuppression in cases of polymorphic PTLD.

## Introduction

Post-transplant lymphoproliferative disorder (PTLD) is a lymphoplasmacytic proliferative disorder that occurs in the setting of hematopoietic stem cells or solid organ transplants. PTLD is divided into non-destructive, polymorphic, monomorphic, and the classic Hodgkin type [1]. Most PTLD cases are B cell type, with less than 15% of cases being T or NK cell type [1,2]. Two-thirds of PTLD cases in adult solid organ transplant recipients are related to Epstein-Barr virus (EBV) [3]. The common presentation of PTLD includes malaise, fever, weight loss, and mononucleosis-like symptoms [1].

Polymorphic PTLD comprises small- to intermediate-sized lymphocytes, immunoblasts, and plasma cells that infiltrate and destroy tissue architecture. They may show malignant features such as nuclear atypia, necrosis, and high mitotic rate [4]. Non-destructive and polymorphic PTLDs tend to regress with a reduction in immune suppression. For more aggressive forms, commonly employed systemic treatments are the rituximab, anti-CD20 monoclonal antibody, with or without combination cytotoxic chemotherapy, and EBV-specific antiviral therapies.

The objective of this study was to examine the demographic factors and treatment modalities that influence survival in patients with polymorphic PTLD.

## Materials and methods

The National Cancer Institute initiated the Surveillance, Epidemiology, and End Results (SEER) database in 1972, which covers around 28% of the United States (US) population. To collect cancer patient data from 2000 to 2018, the SEER*Stat software version 8.4.0 (National Cancer Institute, Maryland, United States) was used while utilizing the International Classification of Diseases Version 3 (ICD-I-3).

No IRB approval was required because the data was from the SEER database, which was publicly available deidentified data and does not require ethical approval.

Eighteen registries from SEER were used to extract data that was exported to SPSS Statistics version 28.0 (IBM Corp. Released 2021. IBM SPSS Statistics for Windows, Version 28.0. Armonk, NY: IBM Corp.) for descriptive analysis. This database contains around 18% of the US population using 18 different registries which are the Alaska Native Tumor Registry, Arizona Indians Tumor Registry, Cherokee Nation Tumor Registry, Connecticut tumor registry, Detroit tumor registry, Georgia Center for Cancer Registry, Greater Bay Area Cancer tumor Registry, Greater California registry, Hawaii Tumor Registry, Iowa Tumor Registry, Kentucky Tumor Registry, Louisiana Tumor Registry, New Jersey Tumor Registry, Seattle-Puget Sound Tumor Registry, and Utah Tumor Registry from SEER software (https://seer.cancer.gov/seerstat/, accessed 5 March 2022).

Demographic factors included age, race, and sex. Clinical data included distant metastases and treatment modality. These variables were collected as they were limited only to those available to the SEER database. Cases that were excluded in this study were cases diagnosed with a “death certificate only” and “autopsy only” and were deemed “unknown."

SPSS Statistics version 28.0 was then used for demographic statistics along with Cox regression analysis, univariate analysis, the multivariable test of associations, the proportional hazard model, and overall survival analysis. A p-value of <.05 was considered significant. Endpoints included overall survival. Multivariate analysis was performed to identify independent risk factors.

## Results

In this study, 332 cases of polymorphic PTLD were identified between 2000 and 2018 using the SEER database.

Demographic characteristics

The median age of the patients was found to be 44 years old. The most common age groups were between the ages of 1-19 (n=100. 30.1%) and 60-69 (n=70. 21.1%). The youngest age in this cohort was less than one year old (Table 1).

Regarding sex, males were the majority in this cohort with 186 (56.0%), while 146 (44.0%) of the cases were female with a female-to-male ratio of 1:1.3. Regarding racial distribution, most cases were White (256, 77.1%), followed by Black (41, 12.3%), and then Asian, Pacific Islander, or Native American (29, 8.7%). Six (1.8%) cases had an unknown race (Table 1).

**Table 1:** Demographic factors of 332 cases of polymorphic PTLD

Variable (n= 332)		Frequency (%)
Age (years)	1-19	100 (30.1%)
	20-29	28 (8.4%)
	30-39	25 (7.5%)
	40-49	26 (7.8%)
	50-59	48 (14.5%)
	60-69	70 (21.1%)
	70-79	30 (9.0%)
	≥80	5 (1.5%)
Sex	Male	186 (56.0%)
	Female	146 (44.0%)
Race	White	256 (77.1%)
	Black	41 (12.3%)
	Asian, Pacific Islander, or American Indian	29 (8.7%)
	Unknown	6 (1.8%)

Treatment characteristics

**Figure 1:**
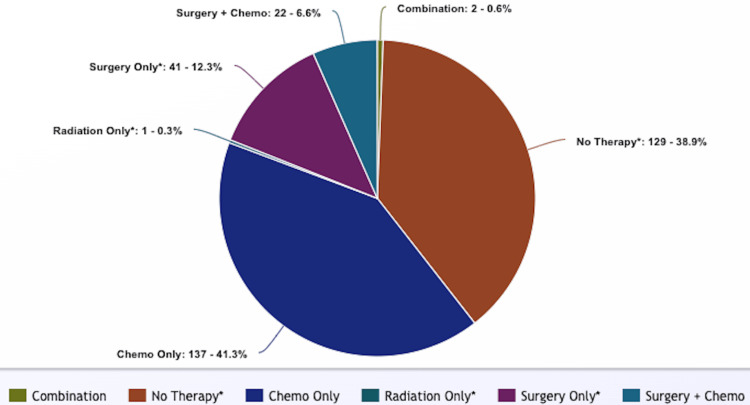
Treatment characteristics of polymorphic PTLD *Chemotherapy status is unknown +Combination: combination of chemotherapy, radiation, and surgery treatment ++Chemotherapy includes all systemic therapies

Of the total cases, 171 (51.5%) cases had an unknown systemic therapy status (referred to as chemotherapy in data description, table, and figures). The majority of the cases in this cohort underwent chemotherapy only (n=137, 41.3%). Forty-one (12.3%) of the cases underwent surgery only with an unknown chemotherapy status. One (0.3%) underwent radiation only with an unknown chemotherapy status. Two (0.6%) cases underwent combination therapy (surgery, radiation, and chemotherapy). One hundred twenty-nine (38.9%) of the cases did not undergo any treatment (Figure 1).

Overall and cause-specific survival by treatment

**Figure 2:**
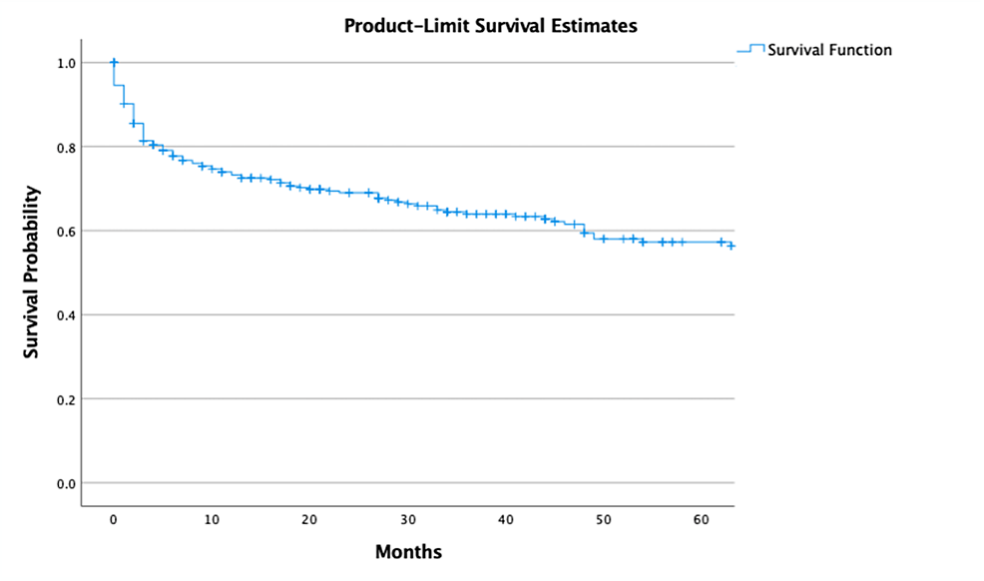
Overall survival of polymorphic PTLD

The overall five-year observed survival was 54.6% (95% CI, 51.1 - 58.1). One-year and five-year survival with chemotherapy only was 63.8% (95% CI, 59.6 - 68.0) and 52.5% (95% CI, 47.7 - 57.3), respectively (Figure 2).

**Figure 3:**
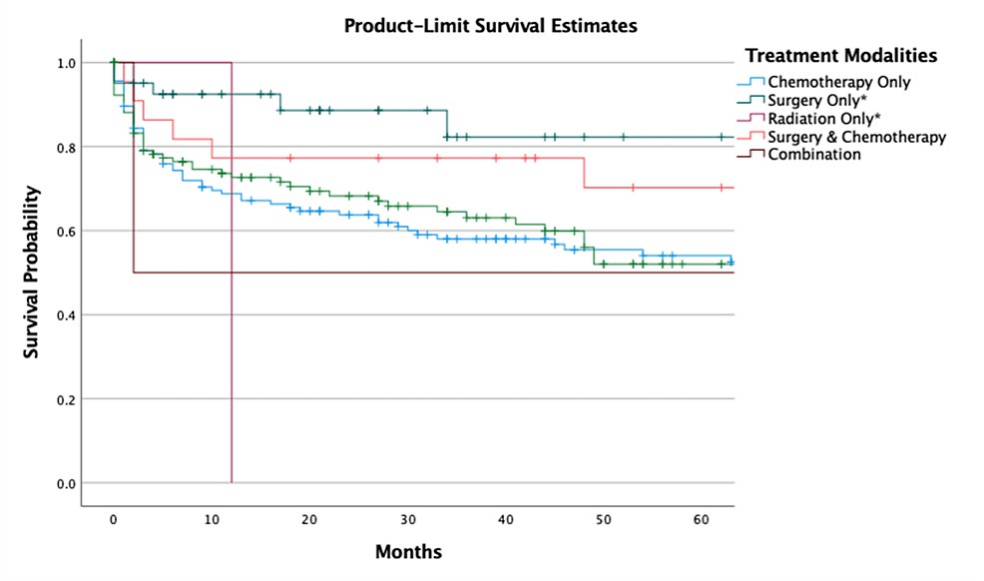
Survival of Different Treatment Modalities of Polymorphic PTLD ++Chemotherapy includes all systemic therapies *Chemotherapy status is unknown

The one-year and five-year survival with surgery as the mainstay of therapy was 87.3% (95% CI, 81.2-93.4) and 60.8% (95% CI, 42.2 - 79.4), respectively. The one-year and five-year without therapy were 67.6% (95% CI, 63.2-72.0) and 49.6% (95% CI, 43.5-55.7), respectively. The survival analysis of radiation alone and other therapies not included in this analysis could not be performed due to the limited number of cases. The Kaplan-Meier analysis of survival modalities is listed (Figure 3).

Survival analysis by race and sex

**Figure 4:**
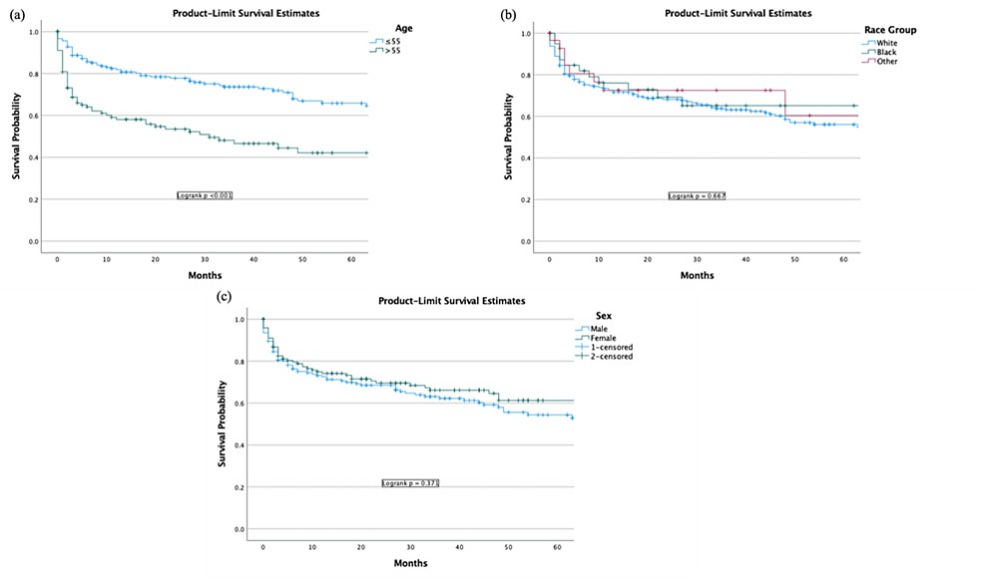
Survival by (a) age, (b) race, and (c) sex of polymorphic PTLD

The one-year survival for white Americans was 67.6% with a 95% CI (95% CI, 64.5-70.7), and at five years, the survival was 52.9% (95% CI, 49.0-56.8). For Black Americans, the one-year survival was 68.8% (95% CI, 60.9-76.7), and at five years, 64.2% (95% CI, 55.6-72.8). For Asian, Indian Americans, and Alaska Natives, the one-year survival was 73.6% (95% CI, 65.0-82.2), and at five years, the survival was 60.2% (46.2-74.2). Race was not a predictor for survival (Figure 4b).

The survival for males at one year was 68.1 % (95% CI, 65.1-71.7), and at five years, the survival was 52.1% (95% CI, 47.5-56.7). The survival for females at one year was 69.1% (95% CI, 65.1-73.1), and at five years, it was 58.0% (95% CI, 52.6-63.4). Sex was not a predictor for survival (Figure 4c).

Multivariate analysis

Multivariate analysis through Cox survival regression analysis identified age >55 years (HR 1.128 (1.139-1.346)), p <0.001 as negative predictors of survival. Univariate analysis revealed that surgery only (HR 0.386 (0.170-0.879), p = 0.023) was a positive predictor for survival (Table 2).

**Table 2:** Univariate and multivariate analysis of independent factors influencing mortality of PTLD * Significant on univariate and multivariate analysis HR: hazard ratio; CI: confidence interval

­		Univariate		Multivariate	
Variables		HR (95% CI)	p-value	HR (95% CI)	p-value
Age	>55 year	1.128 (1.139-1.346)	<0.001*	2.192 (1.522-3.158)	<0.001*
Surgery only	Did not undergo	0.386 (0.170-0.879)	0.023*	0.460 (0.201-1.053)	0.066

## Discussion

PTLD is a rare complication of organ transplantation with an incidence of 2-20%, which can present with varying symptoms depending on the subtype and location. We analyzed a large cohort of patients with polymorphic PTLD to examine the influence of demographic factors and treatments on survival. In this study, we found that the largest cohort of cases was found in pediatric patients. We also found that most patients treated for polymorphic PTLD received systemic therapy, although many did not receive any treatment. We also found that five-year survival was highest in those who underwent surgery. Race and sex did not influence survival, although an age greater than 55 years negatively impacted survival.

Since its first report in 1969, PTLD has been increasingly identified as one of the most serious complications of organ transplantation and, unfortunately, the most common malignancy after solid organ transplantation (excluding non-melanoma skin cancers) [5]. T cell immunosuppression and transplants from an EBV-positive donor to an EBV-negative recipient are key determinants of the risk of EBV-associated PTLD [6]. While the incidence of PTLD in hematopoietic stem cell transplantation without T cell depletion is approximately 1%, the risk is increased by 50-120% in solid organ transplant recipients [1]. Among solid organ transplants, heart, lung, and intestinal transplant recipients are at increased risk than renal or liver transplant recipients [7]. Early-onset PTLD (occurring within two years of kidney transplantation) is usually associated with EBV, and late-onset PTLD (occurring after two years of kidney transplant) is thought to be related to immunosuppression [7]. About one-half to two-thirds of all PTLDs are associated with previous EBV infection [3,8]. Attenuation of T cell control of EBV-infected B cells is the mechanism for PTLD in these patients [1]. Diminished T cell immunosurveillance at the extremes of age due to immaturity or senescence of the immune system, respectively, the immunosuppressive effect of the antirejection regimen, and host genetic factors such as polymorphisms in cytokine genes or certain donor-recipient human leukocyte antigen combinations may help identify the population at risk [1].

T cell depleting therapies such as anti-thymocyte globulin, used in some allogeneic hematopoietic stem cell transplants, and intense immunosuppression, as in heart transplants, pose the highest risk of PTLD [9]. Among calcineurin inhibitors, tacrolimus increases the risk slightly more than cyclosporine [9], while mycophenolate mofetil may not be associated with an increased risk of PTLD [10]. Since PTLDs activate the mammalian target of the rapamycin (mTOR) pathway, sirolimus may even have therapeutic benefits [1].

Most cases of PTLD are of B cell origin (approximately 95%), with the remaining being of T or T/NK cell origin. Most PTLDs of B cells (approximately two-thirds) and some T cell PTLD (approximately 10%) are related to EBV. The World Health Organization recognizes four morphologic variants based on morphologic and immunophenotypic features of PTLD [8]. Monomorphic PTLD is composed of monoclonal malignant B or T lymphoid cells that efface the tissue architecture and meet specific criteria for non-Hodgkin lymphoma subtypes (except mantle cell and follicular subtypes). Classical Hodgkin lymphoma PTLD is the least common variant of PTLD [8]. Non-destructive PTLD is characterized by preserved lymph node architecture and can have benign polyclonal plasmacytic hyperplasia, infectious mononucleosis-like features, or florid follicular hyperplasia. Polymorphic PTLD makes up around 14% of PTLD [11]. Polymorphic PTLD effaces tissue architecture by a pleomorphic lymphoid infiltrate comprised of cells along the spectrum of B cell maturation. Polymorphic PTLD can either be intranodal or extranodal, with lymph node biopsies exhibiting architectural disruption by infiltrating B cells at any range of maturation, and extranodal lesions presenting with local destruction [12]. Polymorphic PTLD was also found to be highly associated with EBV positivity and fewer genetic mutations compared to other subtypes [13].

Adverse prognostic measures in PTLD are older age (>55 years), elevated serum creatinine (>1.5 mg/dL), elevated lactate dehydrogenase, certain sites of disease (central nervous system or serosa involvement), and monomorphic or T cell histology [14]. In our analysis, we found that advanced age negatively affected survival in polymorphic PTLD, which is consistent with previous research in PTLD. However, we found that neither race nor sex predicted survival in polymorphic PTLD [15]. Although a previous study found that females had a better prognosis in PTLD, we found that this difference does not remain when evaluated based on the subtype of PTLD [15].

We also found that the largest percentage of cases of polymorphic PTLD was in the pediatric age group. In line with previous studies, polymorphic PTLD was found to be the predominant subtype in children [6], although some report that the monomorphic subtype is the most common [10]. In a study of pediatric heart transplants, non-destructive and polymorphic PTLD showed higher survival rates than the monomorphic and classical Hodgkin lymphoma subtypes [11]. However, a recent study of adult PTLD patients found no differences in survival between subtypes [16]. Further research is needed into the differences in outcomes based on subtype and age in PTLD.

The highest survival rates for polymorphic PTLD were found in patients who underwent surgery followed by patients who received both systemic therapy and surgery and those who received no treatment. This is complimentary to previous findings that surgery increased survival rates in patients with polymorphic PTLD, compared to the reduction in immunosuppression alone [17]. Reduction in immunosuppression has historically been the first-line treatment for PTLD. In addition, the immunotherapy agent rituximab (if CD20 is positive) and cytotoxic chemotherapy can be used [18]. EBV-targeted antiviral therapies, including acyclovir and ganciclovir, have been used, but their efficacy remains controversial [19]. EBV-specific cytotoxic T cells have shown success when used for hematopoietic stem cell transplant [20]. In addition to these methods, radiation and autologous stem cell transplantation have been employed for selective patients [2]. In recent years, the use of cytotoxic chemotherapy in addition to the reduction of immunosuppression has increased in frequency [16].

Limitations

Limitations in this study include the availability of data in the SEER database used for our retrospective analysis. In the data collection, it may underestimate the true incidence of PTLD in the population [21]. The systemic therapy status was unknown for many patients, and the exact components of systemic therapy regimens (such as cytotoxic chemotherapy, immunotherapy or anti-viral therapy, or a combination of these) were not reported. Analysis of radiation therapy and other therapies could not be performed due to few cases utilizing these treatments. The type of surgery performed data is not available in the SEER database. Data were not analyzed by location of the tumor, type of organ transplant, or EBV positivity status.

## Conclusions

Polymorphic PTLD is a rare complication of organ transplantation that is highly associated with EBV positivity. In our study, it was found that PTLD most often presents in the pediatric age group, and its occurrence in those older than 55 was associated with a worse prognosis. Although the reduction in immunosuppression has typically been the first-line treatment for PTLD, we also analyzed that treatment with surgery was the best predictor for survival in polymorphic PTLD. These findings, along with the increasing use of systemic therapy in PTLD patients, may warrant further research into the best treatment modalities for polymorphic PTLD.
